# Possible New Hepatitis B Virus Genotype, Southeast Asia

**DOI:** 10.3201/eid1411.080437

**Published:** 2008-11

**Authors:** Christophe M. Olinger, Prapan Jutavijittum, Judith M. Hübschen, Amnat Yousukh, Bounthome Samountry, Te Thammavong, Kan Toriyama, Claude P. Muller

**Affiliations:** National Public Health Laboratory/*Centre de Recherche Public****–***Santé, Luxembourg (C.M. Olinger, J.M. Hübschen, C.P. Muller); Chiang Mai University, Chiang Mai, Thailand (P. Jutavijittum, A. Yousukh); National University of Lao, Vientiane, Laos (B. Samountry); Lao Red Cross, Vientiane (T. Thammavong); Nagasaki University, Nagasaki, Japan (K. Toriyama)

**Keywords:** Phylogeny, recombination, genotype, Laos, dispatch

## Abstract

We conducted a phylogenetic analysis of 19 hepatitis B virus strains from Laos that belonged to 2 subgenotypes of a new genotype I. This emerging new genotype likely developed outside Southeast Asia and is now found in mixed infections and in recombinations with local strains in a geographically confined region.

As a result of mutations and recombinations, hepatitis B virus (HBV) has evolved into 8 known genotypes (A–H), with a putative new genotype I recently found in Asia (*1*,*2*). Some genotypes have been associated with distinct clinical patterns, and their detection and identification are important for virus and disease surveillance.

In Laos, 8.7% of the population are chronically infected with HBV, and perinatal transmission is the most common route of infection. Here, we present the phylogenetic analysis of 19 related strains found in voluntary blood donors from Laos that cluster with the new genotype I.

## The Study

**Figure 1:**
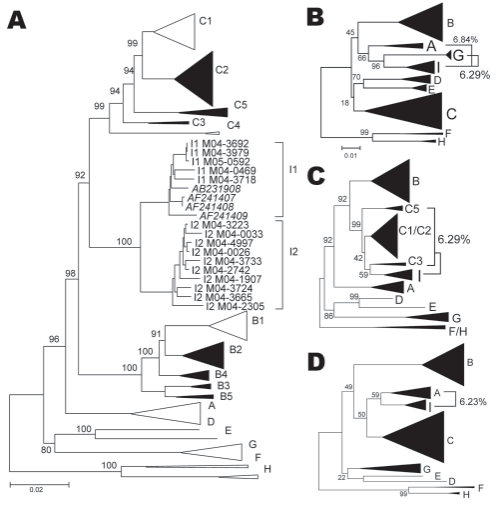
A) Phylogenetic comparison of all complete genotype I genomes (n = 15) obtained and compared to sequences of all known genotypes and subgenotypes. Non–genotype I genotypes identified in Lao People’s Democratic Republic in the present study are shown as full triangles. Numbers indicate bootstrap values of important nodes. B–D) Phylogenetic comparison of positions 400–1400 (left), 1400–3000 (middle), and 3000–400 (right), of all genotype I strains with all known genotypes and subgenotypes. Percentages indicate average genetic distances between genotype I and G, C, or A, respectively. Scale bars indicate number of substitutions per site.

Phylogenetic analysis of sequences obtained from hepatitis B surface antigen–positive first-time blood donors from donation centers in Vientiane City and central provinces of Laos showed that 163 (42.2%) strains were of genotype B, and 204 (55.4%) were of genotype C. Subgenotypes included B2 (18), B3 (1), B4 (128), and B5 (16), as well as C2 (190), C3 (1), and C5 (13) (Figure 1, panel **A**). Nineteen strains, including 15 complete sequences, did not group with any of the known genotypes A–H. These sequences formed 2 clusters, which were emerging from a common node (bootstrap value of 100%; Figure 1, panel **A**). One of the clusters grouped with a recently reported single strain from Vietnam, for which we had previously defined a new genotype I (*2*). The 2 new groups from Laos will be referred to here as subgenotypes I1 and I2. Notably, all I1 strains were of serotype *adw*, whereas all I2 subgenotypes were of serotype *ayw*. With 1 exception, all genotype I strains were found in donors living in Vientiane City. Strains recovered from Hanoi, Vietnam, 8 years ago and reported as aberrant strains (*3*) also group with subgenotype I1.

Detailed analysis of full genome sequences showed that genotype C strains as a group were most closely related to genotype I (average Kimura distance of 7.89%, Table 1). The closest subgenotype was C3 with a 7.0% average Kimura distance (data not shown). The bootstrap value of the separating node was 92% (Figure 1, panel **A**), which is well above the bootstrap value of the G/DE node. On the S gene level, genotype I was most closely related to genotype G with a distance of 4.23% and a bootstrap value of 96% at the separating node (data not shown). Within the 2 subgenotypes I1 and I2, an average diversity over the complete genome of 1.19% and 0.94% was calculated; this difference increased to 2.33% when all strains were considered as a single group. The maximal genetic distance between 2 full-length genotype I strains was 4.3%. All clusterings were verified by maximum likelihood tree construction (data not shown). Thus, in accordance with published criteria (*4*), these values warrant the definition of a new genotype I with 2 subgenotypes I1 and I2.

**Table 1:** Average Kimura distances (in %) within (**boldface**) and between reference complete genome sequences of genotypes A to H and the putative new genotype I and subgenotypes I1 and I2

Genotype	A	B	C	D	E	F	G	H	I
A	***4.26***								
B	10.27	**4.14**							
C	9.95	10.17	**4.79**						
D	10.89	11.84	11.6	**2.95**					
E	10.88	12.33	12	8.2	**1.2**				
F	16.16	16.3	15.98	16.07	15.96	**7.18**			
G	12.48	14.28	14.2	12.46	11.75	17.56	**0.33**		
H	16.58	16.79	16.31	15.96	16.87	9.46	17.37	**2.29**	
I	8.43	9.7	7.89	10.95	11.23	15.74	12.13	16.21	**2.33**
I1	8.37	9.69	7.85	11.03	11.21	15.5	12.1	12.1	
I2	8.49	9.72	7.93	10.89	11.26	15.94	12.16	12.16	

**Figure 2:**
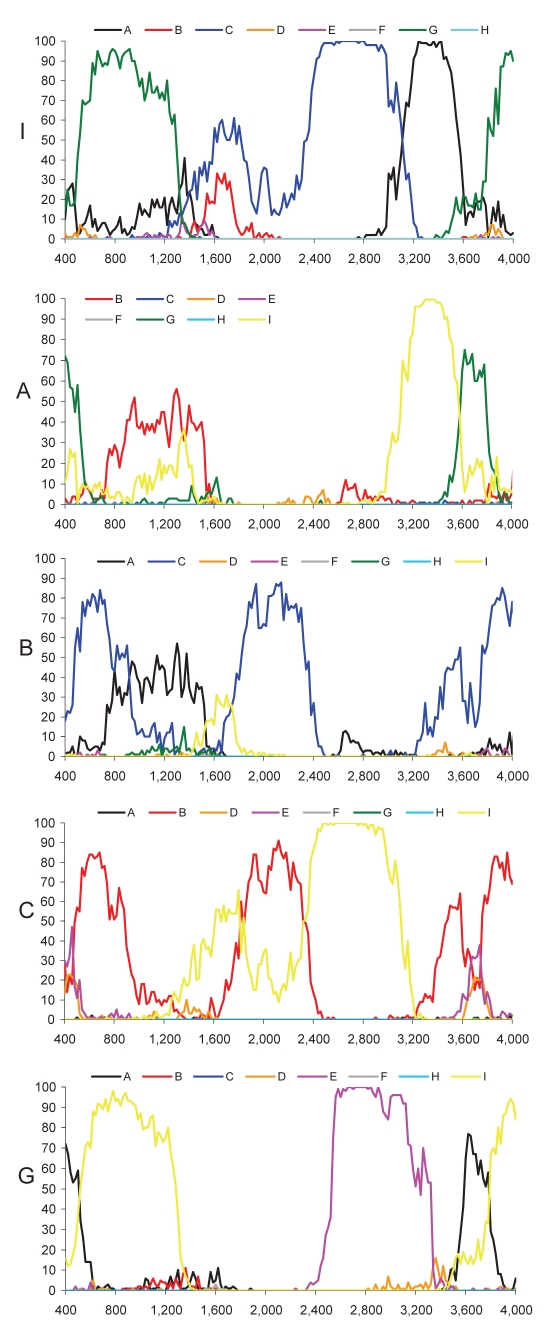
Bootscan analysis of genotypes I, A, B, C, and G compared with genotypes A–H. Data points correspond to the center of sequence windows of 800 bp. For the analysis of the first 400 nt, the beginning of the genome was duplicated at the end of the sequence: nt 3200–4000 represent positions 0–800.

Most current genotypes of HBV seem to be the result of 1 or several recombination events (*5*). In particular, this is evident for the B/C recombinant, which has spread in mainland Asia (*6*) and has been defined as genotype Ba. Also genotypes B and C show some similarity with each other (Figure 2). Bootscan analysis (*7*) of all genotype I strains, including M04–3665, amplified by complete genome PCR, showed similarities with genotype C (nt 1400–3000), A (nt 3000–400), and G (nt 400–1400) by using a window size of 800 nt (Figure 2). Smaller bootscan windows tended to blur the relatedness. Phylogenetic reconstruction (Figure 1, panels **B**–**D**) and BLAST searches (http://blast.ncbi.nlm.nih.gov) (Table 2) of the above fragments of genotype I sequences confirmed the results of the bootscan analysis, which suggests that this genotype may also have evolved from a series of recombination events in a distant past.

**Table 2:** Characteristics of strains most closely related to different recombinant fragments of genotype I (M04-0469): genotypes and genetic distances

Characteristic	nt 400–1400	d	nt 1400–3000	d	nt 3000–400	d
Most similar genotype	G	6.29*	C	6.29	A	6.23
2nd most similar genotype	A	6.84	B	9.14	C	8.61
Most similar subgenotype	NA		C3	5.42	A4	5.23
Most similar strain	G, AF160501	5.51	C3, X75656	4.1	A3, AM184126	4.5
2nd most similar strain	G, AB056513	5.62	C1,AB222715	4.3	A4, AY934764	4.7

*Distances (in %) to genotypes of subgenotypes are average distances, that is, the A-like sequence was in average most similar to subgenotype A4 but the minimal distance observed was to an A3 strain. nt, nucleotide; d, distance; NA, not applicable.

The genotype C–like fragment was most closely related to subgenotype C3 (Table 2), thus far found only in the Pacific, except for a single incomplete strain in Laos (from this study). C1, found in Japan, South Korea, the People’s Republic of China, and Uzbekistan (*8**,**9*), is the second most closely related subgenotype. The genotype A–like fragment was most similar to subgenotypes A3 and A4, recently found by us and confirmed by others in sub-Saharan Africa (*10*–*12*). The apparent relatedness with the defective genotype G is even more surprising, since it has only been found sporadically in the United States, Japan, Germany, and France (*13*–*15*). Thus, none of the contributing genotypes or subgenotypes have ever been identified in Southeast Asia, which evokes questions about the origin of genotype I in this region.

To further exclude artifacts of mixed infections, various genome regions of 15 genotype I strains were cloned. The 92 clones analyzed clustered with the same group as the uncloned parent sequences. In just 2 cases, for which only the preS/S gene was available for analysis (M05–0659 and M04–2769), a mixed infection with several genotypes was found. The first (M05–0659) contained an I1 and I1/B4 recombinant sequence (B4 on the last 300 nt), while the second (M04–2769) contained 5 different species: 1 B5/C2 recombinant (C2 on the last 400 nt), 1 C5/C2 recombinant (C2 on the last 400 nt), 1 genotype C2 sequence, 4 genotype C5 sequences, and 1 C/I2 recombinant (C subgenotype unclear; I2 on nt 200–600).

Four strains with no signs of mixed infections in the sequence electropherograms clustered inconclusively in the phylogenetic analysis of partial sequences (data not shown). These strains were cloned and identified by bootscan analysis as recombinants between several genotypes and/or subgenotypes. In 3 recombinants, only subgenotypes I1 and I2 were involved (M04–3739, M04–2531, and M04–0309). The 2 latter recombinants had identical bootscan patterns (I1 between nt 1400–1900), while in the first recombinant, the I2 sequence was shifted downstream by 200 nt (data not shown). Strain C04–0790 showed a similar pattern to M04–3739, but the I1 sequence was replaced by a C2 sequence. Bootscan analysis of a previously reported strain (GenBank/EMBL/DDBJ no. AB231909) showed a B4/I1 recombinant with the I1 sequence identified between positions 600 and 1864. The most similar strains for each of the distinct regions of these recombinants are virtually all circulating in Laos (this study). In fact, the C2 sequence of the C04–0790 recombinant strain was identical to the C04–1257 strain found in a different district of Vientiane. This strain could have been a potential donor strain.

## Conclusions

In a preliminary report, we discussed the need to assign a new genotype (I) to strains that we had found in Laos (*1*). More recently, Tran et al. defined a new genotype I on the basis of a single, similar strain from Vietnam (*2*). Here we analyzed a larger number of new sequences which formally comply with the definition of a new genotype (I) and 2 subgenotypes of it. A complex recombination pattern suggests that genotype I was formed by recombinations outside of Southeast Asia before spreading within Laos and Vietnam, and giving birth to a new genotype with subgenotypes, which later recombined with regional strains. Identification and analysis of genotype I strains provide further evidence of the importance of recombination in the evolution and genesis of new HBV genotypes, a complexity not fully acknowledged by the current genotype classification. Nucleotide sequences from this study have been submitted to international public databases under accession nos. FJ023546–48, FJ023553–60, FJ023566–68, FJ023572–73, FJ023577–630, FJ023642, FJ023659–83, FJ023700–07, FJ023854, FJ023878–85, FJ023936, FJ023968, FJ023994, and FJ358584–98.

## References

[1] Jutavijittum P, Olinger CM, Hübschen J, Yousukh A, Samountry B, Thammavong T, Molecular phylogeny of hepatitis B virus in Laos reveals multiple subtypes of endemic genotypes, numerous unclassifiable strains and a growing prevalence of recombinations within sub- and genotypes and a potential new genotype. In: Proceedings of the Molecular Biology of Hepatitis B Viruses, Rome, Italy, 2007.

[2] Tran TT, Trinh TN, Abe K. New complex recombinant genotype of hepatitis B virus identified in Vietnam. J Virol. 2008;82:5657–63. 10.1128/JVI.02556-07 18353958 PMC2395174

[3] Hannoun C, Norder H, Lindh M. An aberrant genotype revealed in recombinant hepatitis B virus strains from Vietnam. J Gen Virol. 2000;81:2267–72. 10950984 10.1099/0022-1317-81-9-2267

[4] Kramvis A, Kew MC. Epidemiology of hepatitis B virus in Africa, its genotypes and clinical associations of genotypes. Hepatol Res. 2007;37(s1):S9–19. 10.1111/j.1872-034X.2007.00098.x 17627641

[5] Simmonds P, Midgley S. Recombination in the genesis and evolution of hepatitis B virus genotypes. J Virol. 2005;79:15467–76. 10.1128/JVI.79.24.15467-15476.2005 16306618 PMC1316029

[6] Sugauchi F, Kumada H, Sakugawa H, Komatsu M, Niitsuma H, Watanabe H, Two subtypes of genotype B (Ba and Bj) of hepatitis B virus in Japan. Clin Infect Dis. 2004;38:1222–8. 10.1086/382885 15127332

[7] Lole KS, Bollinger RC, Paranjape RS, Gadkari D, Kulkarni SS, Novak NG, Full-length human immunodeficiency virus type 1 genomes from subtype C-infected seroconverters in India, with evidence of intersubtype recombination. J Virol. 1999;73:152–60. 9847317 10.1128/jvi.73.1.152-160.1999 PMC103818

[8] Kim H, Jee YM, Song BC, Shin JW, Yang SH, Mun HS, Molecular epidemiology of hepatitis B virus (HBV) genotypes and serotypes in patients with chronic HBV infection in Korea. Intervirology. 2007;50:52–7. 10.1159/000096313 17164558

[9] Norder H, Courouce AM, Coursaget P, Echevarria JM, Lee SD, Mushahwar IK, Genetic diversity of hepatitis B virus strains derived worldwide: genotypes, subgenotypes, and 10.1159/000080872 15564741

[10] Makuwa M, Souquiere S, Telfer P, Apetrei C, Vray M, Bedjabaga I, Identification of hepatitis B virus subgenotype A3 in rural Gabon. J Med Virol. 2006;78:1175–84. 10.1002/jmv.20678 16847965

[11] Mulders MN, Venard V, Njayou M, Edorh AP, Bola Oyefolu AO, Kehinde MO, Low genetic diversity despite hyperendemicity of hepatitis B virus genotype E throughout West Africa. J Infect Dis. 2004;190:400–8. 10.1086/421502 15216479

[12] Olinger CM, Venard V, Njayou M, Oyefolu AO, Maiga I, Kemp AJ, Phylogenetic analysis of the precore/core gene of hepatitis B virus genotypes E and A in West Africa: new subtypes, mixed infections and recombinations. J Gen Virol. 2006;87:1163–73. 10.1099/vir.0.81614-0 16603517

[13] Chudy M, Schmidt M, Czudai V, Scheiblauer H, Nick S, Mosebach M, Hepatitis B virus genotype G monoinfection and its transmission by blood components. Hepatology. 2006;44:99–107. 10.1002/hep.21220 16799987

[14] Kato H, Orito E, Gish RG, Sugauchi F, Suzuki S, Ueda R, Characteristics of hepatitis B virus isolates of genotype G and their phylogenetic differences from the other six genotypes (A through F). J Virol. 2002;76:6131–7. 10.1128/JVI.76.12.6131-6137.2002 12021346 PMC136184

[15] Shibayama T, Masuda G, Ajisawa A, Hiruma K, Tsuda F, Nishizawa T, Characterization of seven genotypes (A to E, G and H) of hepatitis B virus recovered from Japanese patients infected with human immunodeficiency virus type 1. J Med Virol. 2005;76:24–32. 10.1002/jmv.20319 15779062

